# Short-lived long non-coding RNAs as surrogate indicators for chemical exposure and LINC00152 and MALAT1 modulate their neighboring genes

**DOI:** 10.1371/journal.pone.0181628

**Published:** 2017-07-18

**Authors:** Hidenori Tani, Sayaka Okuda, Kaoru Nakamura, Motohide Aoki, Tomonari Umemura

**Affiliations:** 1 Environmental Management Research Institute, National Institute of Advanced Industrial Science and Technology (AIST), 16–1, Onogawa, Tsukuba, Ibaraki, Japan; 2 Department of Molecular Life Sciences, School of Life Sciences, Tokyo University of Pharmacy and Life Sciences, 1432–1 Horinouchi, Hachioji, Tokyo, Japan; Universitat des Saarlandes, GERMANY

## Abstract

Whole transcriptome analyses have revealed a large number of novel long non-coding RNAs (lncRNAs). Although accumulating evidence demonstrates that lncRNAs play important roles in regulating gene expression, the detailed mechanisms of action of most lncRNAs remain unclear. We previously reported that a novel class of lncRNAs with a short half-life (*t*_1/2_ < 4 h) in HeLa cells, termed short-lived non-coding transcripts (SLiTs), are closely associated with physiological and pathological functions. In this study, we focused on 26 SLiTs and nuclear-enriched abundant lncRNA, MALAT1(*t*_1/2_ of 7.6 h in HeLa cells) in neural stem cells (NSCs) derived from human induced pluripotent stem cells, and identified four SLiTs (TUG1, GAS5, FAM222-AS1, and SNHG15) that were affected by the following typical chemical stresses (oxidative stress, heavy metal stress and protein synthesis stress). We also found the expression levels of LINC00152 (*t*_1/2_ of 2.1 h in NSCs), MALAT1 (*t*_1/2_ of 1.8 h in NSCs), and their neighboring genes were elevated proportionally to the chemical doses. Moreover, we confirmed that the overexpression of LINC00152 or MALAT1 upregulated the expressions of their neighboring genes even in the absence of chemical stress. These results reveal that LINC00152 and MALAT1 modulate their neighboring genes, and thus provide a deeper understanding of the functions of lncRNAs.

## Introduction

Mammalian transcriptome analyses have revealed the presence of thousands of unannotated long non-coding RNAs (lncRNAs) with distinct transcriptional units [1]. lncRNAs are defined as RNA molecules of greater than 200 nucleotides in length that do not contain any apparent protein-coding potential, as determined largely by bioinformatics [2]. The majority of lncRNAs are transcribed by RNA polymerase II (Pol II) occupancy, histone modifications related to transcription initiation and elongation, and polyadenylation [3]. There is increasing evidence of lncRNAs participating in diverse biological processes by functioning as signals, decoys, guides, and scaffolds [4]. lncRNAs also exhibit cell type-specific expression and respond to diverse stimuli, suggesting that their expression is under considerable transcriptional control. Furthermore, lncRNAs can serve as molecular signals because transcription of individual lncRNAs occurs at very specific times and places to integrate developmental cues, interpret cellular context, and respond to diverse stimuli [5]. Although the importance of lncRNAs has been documented in recent years, the biological and physiological functions of many lncRNAs remain largely unknown.

The steady-state RNA levels of a gene are determined by regulation at multiple levels, including transcriptional initiation, elongation, splicing, export, and degradation. Transcriptional regulation and RNA degradation are independently regulated [6]. RNA stability is a particularly important determinant of eventual transcript levels [7]. To measure genome-wide lncRNA stability, we previously developed a novel method called 5-bromouridine immunoprecipitation chase assay (BRIC) or BRIC through deep sequencing (BRIC-Seq) [8]. BRIC-Seq has revealed 785 lncRNAs with short half-lives (*t*_1/2_ < 4 h), termed short-lived non-coding transcripts (SLiTs) [8].

SLiTs include known regulatory lncRNAs, such as HOX transcript antisense RNA (HOTAIR) [9] and cyclin dependent kinase inhibitor 2B antisense RNA 1 (CDKN2B-AS1)/antisense noncoding RNA in the inhibitors of cyclin dependent kinase 4 locus (ANRIL) [10], which are involved in the regulation of gene expression through epigenetic modification; nuclear enriched abundant transcript 1 variant 2 (NEAT1_v2) [1,11], an architectural RNA component of paraspeckle that is involved in the innate immune response via transcriptional regulation of antiviral genes; taurine up-regulated 1 (TUG1) [12], which interacts with enhancer of zeste 2 polycomb repressive complex 2 subunit (EZH2) and represses cell cycle genes; and growth arrest specific 5 (GAS5) [13], which acts as a decoy hormone response element for the glucocorticoid receptor, thereby blocking the upregulation of gene expression by the activated glucocorticoid receptor. Recently, the physiological functions of long intergenic non-protein coding RNA 00152 (LINC00152) and metastasis associated lung adenocarcinoma transcript 1 (MALAT1) have been reported. Levels of LINC00152, also known as cytoskeleton regulator RNA (CYTOR), have been shown to be increased in gastric cancer tissue [14]. LINC00152 expression levels in gastric carcinoma were significantly increased compared with in matched normal tissue and normal mucosa from healthy controls. LINC00152 also acts as a novel biomarker in predicting diagnosis of hepatocellular carcinoma [15]. MALAT1 is stably retained in the nucleus and specifically localized to nuclear speckles (subnuclear structures enriched for pre-mRNA splicing factors) [16,17]; however, depletion of MALAT1 does not affect the localization on nuclear speckle markers [18]. MALAT1 overexpression enhanced RNA pol (II), P300 and cell-cycle alteration and expression-elevated protein in tumour (CREPT) loading on the promoter region of telomere repeat-binding factor 2 (TRF2), triggering the overexpression, phosphorylation and small ubiquitin-like modifier (SUMO)ylation of TRF2 [19]. Moreover, we identified six SLiTs (CDKN2B-AS1, MIR22 host gene (MIR22HG), GA binding protein transcription factor beta subunit 1 antisense RNA 1 (GABPB1-AS1), long intergenic non-protein coding RNA 1184 (LINC01184), LINC00152, and long intergenic non-protein coding RNA 541471 variant 2 (LINC0541471_v2)) that rapidly and extensively responded to chemical stresses in human induced pluripotent stem cells (iPSCs) [20]. Thus, as-yet unidentified SLiTs have the potential to be novel regulatory lncRNAs.

Here, we sought to identify novel SLiTs that respond to four typical hazardous chemicals (hydrogen peroxide, mercury II chloride, zinc chloride, or cycloheximide) in neural stem cells (NSCs) derived from iPSCs. We also used NSCs derived from iPSCs because they are not genetically altered unlike immortalized cells, and iPSCs have the ability to undergo numerous cycles of cell division while maintaining their cellular identity. Thus we considered that NSCs derived from iPSCs are a good model for cell-based assessment of toxicants. We identified many SLiTs that responded generally or specifically to chemical stresses in NSCs. We also report that LINC00152 and MALAT1 modulated their neighboring genes in response to chemical stress.

## Materials and methods

### Chemicals

Hydrogen peroxide, mercury II chloride, and zinc chloride were purchased from Wako, Japan. Cycloheximide was purchased from Biovision, USA. These chemicals were dissolved in dimethyl sulfoxide (Wako) and diluted in culture medium at 0.1% vol/vol final concentration.

### Cell culture

The human iPSC line 201B7 was provided by the RIKEN BioResource Center in Japan. This iPSC line was derived from human dermal fibroblasts from the facial dermis of 36-year old Caucasian female [21]. The 201B7 iPSCs were maintained in primate embryonic stem (ES) cell medium (ReproCELL, Japan) supplemented with 4 ng/mL recombinant human fibroblast growth factor (FGF)-basic (146 aa), and penicillin–streptomycin (Life Technologies, USA) on mitomycin C (Kyowa kirin, Japan)-treated mouse embryonic fibroblasts (SNL 76/7; DS Pharma Biomedical, Japan) as feeder cells. These cultures were maintained at 37°C in a humidified incubator with 5% CO_2_. The iPSCs were then cultured with an mTeSR1 medium kit (Stem Cell Technologies, Canada) on Matrigel hESC-qualified matrix (BD, USA) without feeder cells. Subsequently, the iPSCs were induced to form NSCs by treatment with PSC neural induction medium (Thermo Fisher Scientific, USA) for 7 days according to the manufacturer’s instructions.

### Immunocytochemistry

To visualize common hNSC markers, we used a human NSC immunocytochemistry kit (Life Technologies). The primary antibodies used were anti-NESTIN mouse monoclonal antibody (Thermo Fisher Scientific; A24345) and anti-SOX1 goat monoclonal antibody (Thermo Fisher Scientific; A24347). The secondary antibodies used were Alexa Fluor 488 donkey anti-mouse (Thermo Fisher Scientific; A24350) and Alexa Fluor 488 donkey anti-goat (Thermo Fisher Scientific; A24349). Fluorescent images were obtained using an Axio Observer Z1 (Zeiss).

### Reverse transcription-quantitative real-time polymerase chain reaction (RT-qPCR)

Total RNA was extracted from cells with RNAiso Plus (TaKaRa, Japan) according to the manufacturer’s instructions. The isolated RNA was reverse transcribed into cDNA using PrimeScript RT Master Mix (Perfect Real Time; TaKaRa). The resulting cDNA was amplified using the primer sets listed in S1–S3 Tables with glyceraldehyde-3-phosphate dehydrogenase (GAPDH), actin beta (ACTB), hypoxanthine phosphoribosyltransferase 1(HPRT1), and phosphoglycerate kinase 1 (PGK1) mRNA levels used for normalization. Relative RNA quantities were calculated as treated values normalized to untreated values. THUNDERBIRD SYBR qPCR mix (Toyobo, Japan) was used according to the manufacturer’s instructions. RT-qPCR analysis was performed using a MyiQ2 (BIO-RAD, USA).

### 5-Ethynyluridine (EU) pulse labeling

Analysis of RNA transcription and degradation rates was conducted by EU pulse-labeling of RNA using a Click-iT Nascent RNA Capture kit (Life Technologies) [22] according to the manufacturer’s instructions, with some modifications. To assess transcription rates, we added EU (400 μM) and the chemicals of interest to the culture medium and incubated the cells for 2 h. The cells were then harvested. Total RNA was isolated using RNAiso Plus (Takara). EU-labeled RNAs were biotinylated and captured using the Click-iT Nascent RNA Capture kit (Life Technologies). To elute the EU-RNAs, magnetic beads were resuspended in 100 μl of buffer A (10 mM Tris–HCl, pH 7.4 and 6.25 mM EDTA). ISOGEN LS (300 μl; Nippon Gene, Japan) was added to the mixture, and EU-labeled RNAs were isolated according to the manufacturer’s instructions; these isolated RNAs were used in the subsequent RT-qPCR assays. The total amount of EU-labeled RNA captured was divided by the input RNA amount. To assess degradation rates, we added EU (200 μM) to the culture medium and incubated the cells for 2 h. The EU-containing medium was then replaced with EU-free medium containing the chemicals of interest, and cells were harvested at the indicated time points. Total RNA was isolated using RNAiso Plus (Takara). EU-labeled RNAs were biotinylated and captured using the Click-iT Nascent RNA Capture Kit (Life Technologies). To elute the EU-RNAs, magnetic beads were resuspended in 100 μl of buffer A (10 mM Tris–HCl, pH 7.4 and 6.25 mM EDTA). ISOGEN LS (300 μl; Nippon Gene, Japan) was added to the mixture, and EU-labeled RNAs were isolated according to the manufacturer’s instructions; these isolated RNAs were used in the subsequent RT-qPCR assays.

### Plasmid construct

A LINC00152 (1–804) expression vector was constructed by subcloning the full-length LINC00152 sequence lacking a poly A tail (based on the LINC00152 sequence, NR_024204 in NCBI). The LINC00152 cDNA was amplified from total RNA purified from human cells, and then cloned into pcDNA3.1 (+) (Invitrogen, USA). Additional information on vector construction can be provided upon request. The MALAT1 expression vector was kindly donated by Dr. Hirose, Hokkaido University, Japan.

### Overexpression of lncRNAs

The expression vector, at 1 mg/mL, was transfected into cells using Lipofectamine 2000 (Invitrogen), according to the manufacturer’s instructions. Cells were harvested 48 h after transfection and total RNAs were isolated using RNAiso Plus (Takara), according to the manufacturer’s instructions. Overexpression levels were determined by RT-qPCR.

## Results

### Human pluripotent stem cell differentiation

**Fig 1:**
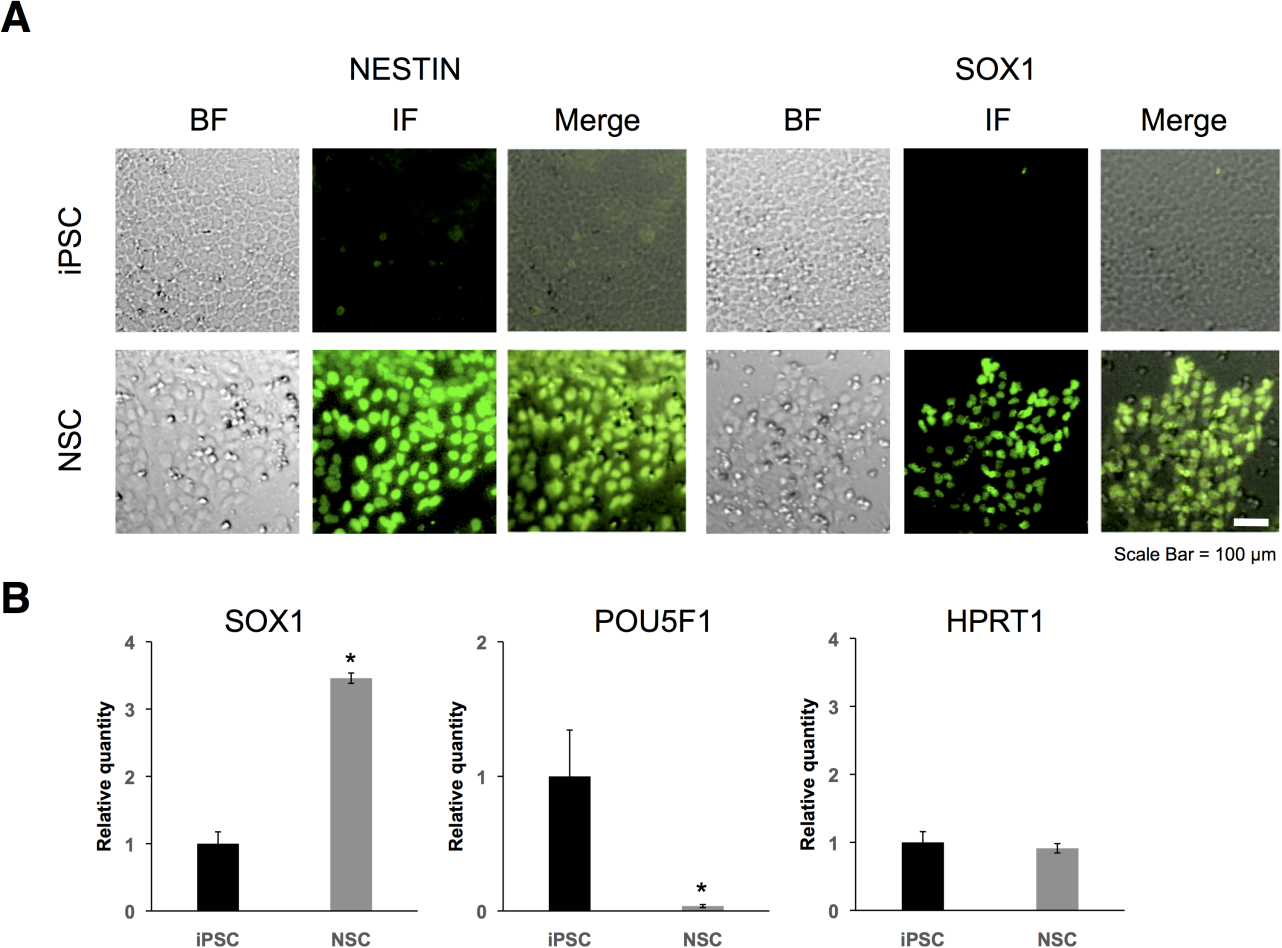
Immunostaining and RT-qPCR of neural stem cell (NSC) markers. (A) Fluorescence images were obtained using a human NSC immunocytochemistry kit (Life Technologies). Cells were immunostained for NESTIN (green; left) and SOX1 (green; right). BF indicated bright field and IF indicated immunofluorescence. (B) Expression levels of SOX1, POU5F1, and HPRT1 in iPSCs (black bar) and in NSCs (gray bar) were determined by RT-qPCR. GAPDH, ACTB, HPRT1, and PGK1 were used for normalization. Values represent mean ± SD obtained from three independent experiments (*P < 0.05, Student’s t test).

We first verified induction of NSCs from iPSCs by immunostaining for the NSC markers NESTIN and SRY-box 1 (SOX1). Previous study showed that NSCs expressed NESTIN and SOX1 as the neural stem cell markers, and did not expressed POU class 5 homeobox 1 (POU5F1) [23]. NSCs are positive for NESTIN and SOX1 (Fig 1A). We also verified induction using RT-qPCR for SOX1, POU5F1 and hypoxanthine phosphoribosyltransferase 1 (HPRT1). HPRT1 is a housekeeping gene and no change was expected to be observed in its expression level. SOX1 mRNA levels were higher in the NSCs than in the iPSCs, and POU5F1 mRNA levels were lower in the NSCs than in the iPSCs (Fig 1B). In contrast, HPRT1 expression levels did not change during induction (Fig 1B). These results indicated that the iPSCs were effectively induced to form NSCs.

### LncRNAs are more useful than mRNAs as surrogate indicators of cellular stress

**Fig 2:**
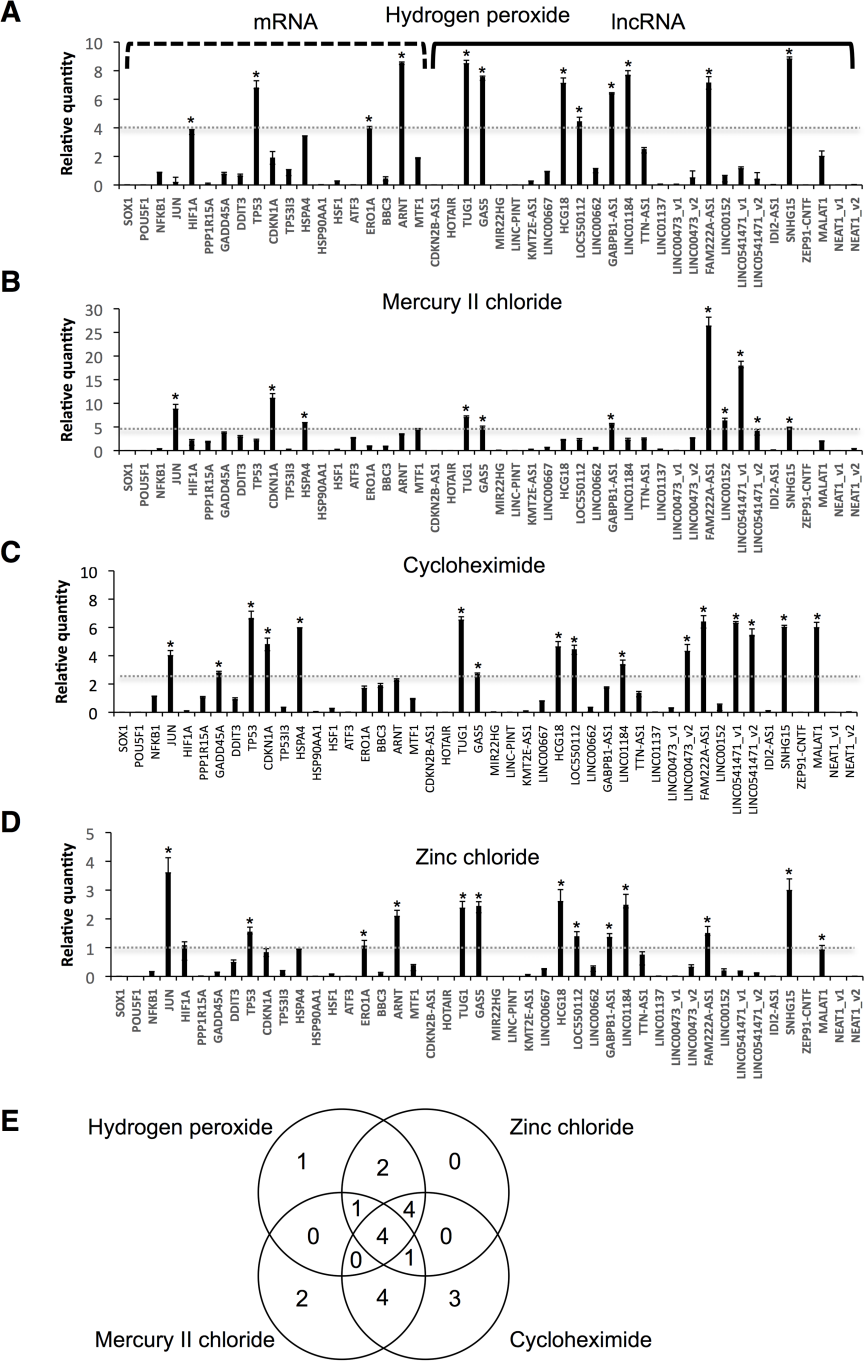
Alterations in mRNA and lncRNA expression levels in NSCs in response to four chemical stressors. NSCs were treated with (A) 100 μM hydrogen peroxide, (B) 100 μM mercury II chloride, (C) 100 μM cycloheximide, or (D) 100 μM zinc chloride for 24 h. Expression levels of the indicated RNAs were determined by RT-qPCR. GAPDH, ACTB, HPRT1, and PGK1 were used for normalization. Values represent mean ± SD obtained from four independent experiments. Y-axis indicated that the expression levels of treated-cells was divided by the those of untreated-cells. Thus, zero indicated the RNA not detectable and one indicated the there was no change in expression levels comparing the untreated-cells. Gray dotted lines indicated cut-off values. Values represent mean ± SD obtained from four independent experiments (*P < 0.05, Student’s t test) over cut-off values. (E) Venn diagram of up-regulated genes by four chemical stressors.

We selected 26 SLiTs and nuclear-enriched abundant lncRNA, MALAT1(*t*_1/2_ of 7.6 h in HeLa cells) [8]. We applied these data for NSC in this study. All of the lncRNAs selected were longer than 200 nt, thus fulfilling the established criterion for lncRNA classification. We examined alterations in the expression levels of the 27 lncRNAs, and of 17 mRNAs that are known as stress biomarkers, following treatment of NSCs with four typical stressors (hydrogen peroxide, mercury II chloride, zinc chloride, or cycloheximide) (Fig 2). Hydrogen peroxide induces oxidative stress, mercury II chloride and zinc chloride induce heavy metal stress, and cycloheximide inhibits translation. The mRNA biomarkers measured were of genes associated with oxidative stress response [nuclear factor kappa B subunit 1 (NFKB1), Jun proto-oncogene, AP-1 transcription factor subunit (JUN); hypoxia inducible factor 1 alpha subunit (HIF1A); DNA damage [protein phosphatase 1 regulatory subunit 15A (PPP1R15A), growth arrest and DNA damage inducible alpha (GADD45A), DNA damage inducible transcript 3 (DDIT3), tumor protein p53 (TP53), cyclin dependent kinase inhibitor 1A (CDKN1A), and tumor protein p53 inducible protein 3 (TP53I3); heat shock response [heat shock protein family A member 4 (HSPA4), heat shock protein 90 alpha family class A member 1 (HSP90AA1) and heat shock transcription factor 1 (HSF1)), endoplasmic reticulum (ER) stress [activating transcription factor 3 (ATF3), endoplasmic reticulum oxidoreductase 1 alpha (ERO1A), and BCL2 binding component 3 (BBC3)]; hypoxia inducible factors [aryl hydrocarbon receptor nuclear translocator (ARNT)]; and heavy metal stress [metal regulatory transcription factor 1 (MTF1)].

First screening was performed with 100 μM chemical treatments for 24 h according to the previous report [24] as follows. Up-regulated genes in chemical responses were easy to measuring differences and highly and rapidly respond as potential surrogate indicators for chemical stresses; therefore, we only focused on up-regulated genes in chemicals stressors. After treatment with 100 μM hydrogen peroxide, NSCs exhibited significantly increased expression levels of the lncRNAs TUG1, GAS5, HLA complex group 18 (HCG18), LOC550112, GABPB1-AS1, LINC01184, family with sequence similarity 222 member A antisense 1 (FAM222-AS1), and small nucleolar RNA host gene 15 (SNHG15) as indicated the cut-off lines (Fig 2A). Treatment with 100 μM mercury II chloride led to significant increases in the expression levels of TUG1, GAS5, GABPB1-AS1, FAM222-AS1, LINC00152, LINC0541471_v1, and SNHG15 (Fig 2B). Treatment with 100 μM cycloheximide resulted in significant increases in the expression levels of the lncRNAs TUG1, GAS5, HCG18, LOC550112, LINC01184, long intergenic non-protein coding RNA 473 variant 2 (LINC00473_v2), FAM222-AS1, LINC0541471_v1, LINC0541471_v2, SNHG15, and MALAT1 (Fig 2C). Treatment with 100 μM zinc chloride increased expression levels of the lncRNAs TUG1, GAS5, HCG18, LOC50112, GABPB1-AS1, LINC01184, FAM222-AS1, and SNHG15 (Fig 2D). In contrast, few of the mRNAs exhibited high expression levels, compared with the expression levels of lncRNAs. These results indicate that SLiTs exhibited a substantial response to chemical stresses. In particular, TUG1, GAS5, FAM222A-AS1, and SNHG15 responded to all four stressors investigated (Fig 2). Venn diagram was represented in Fig 2E. These quantification results were indicated in S4–S7 Tables.

### RNA transcription and degradation rates

**Fig 3:**
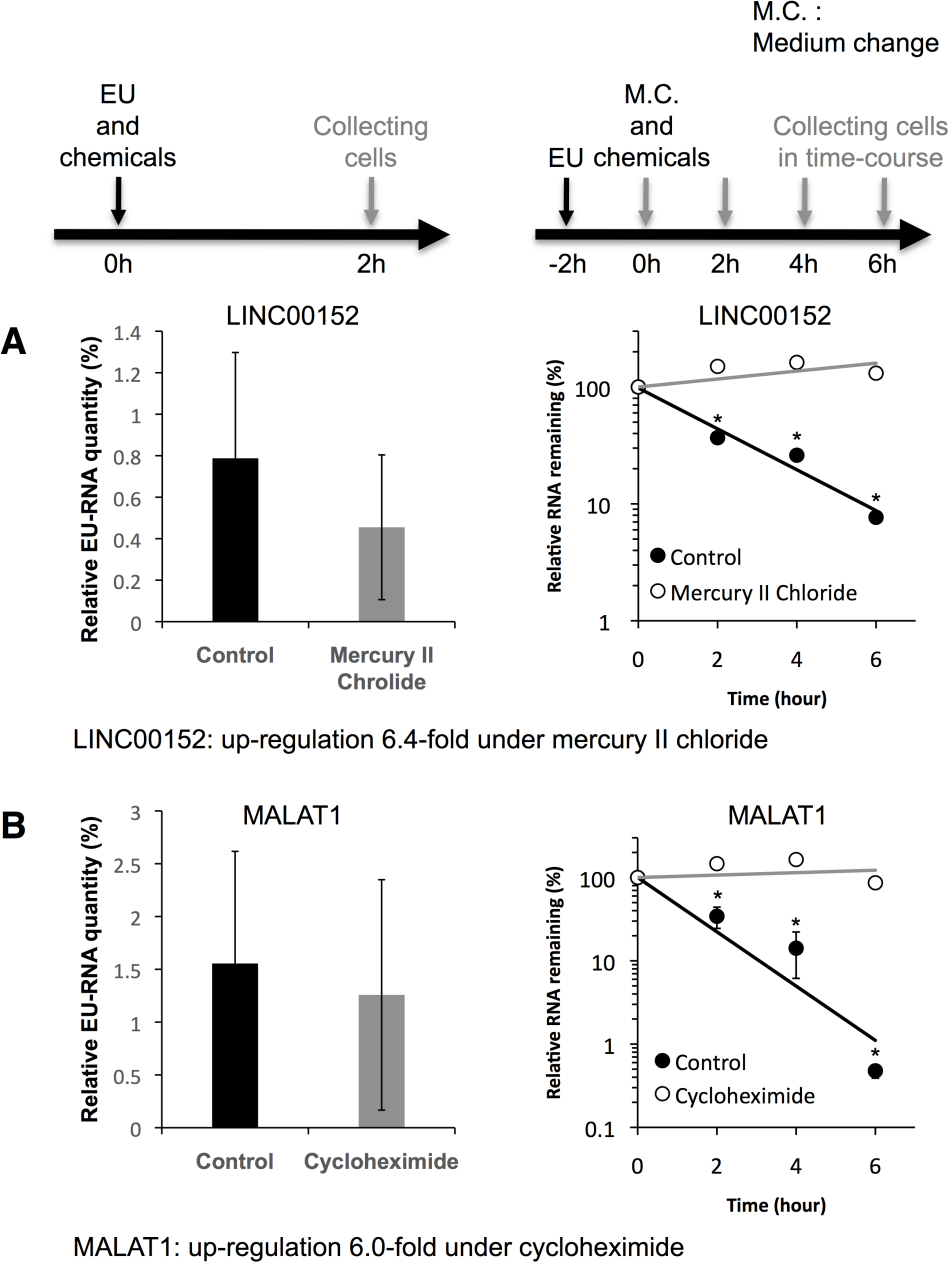
Chemical stresses prolonged the decay rates of LINC00152 and MALAT1, but did not affect transcription rates. The transcription rates of (A) LINC00152 and (B) MALAT1 were determined in control cells (black bar) and chemical-stressed cells (gray bar). Nascent LINC00152 and MALAT1 were transcribed with the incorporation of EU. Relative EU-RNA quantity indicated the total amount of EU-labeled RNA captured was divided by the input RNA amount, and indicated the transcription rate. The decay rates of (A) LINC00152 and (B) MALAT1 were determined in control cells (solid circle and black bar) and in chemical-stressed cells (open circle and gray bar). Values represent mean ± SD obtained from three independent experiments (*P < 0.05, Student’s t test).

Recent studies have revealed that LINC00152 and MALAT1 are prominently involved in biological functions and are sensitive to chemical stresses (Fig 2) [14,15,25–29]. Moreover, these lncRNAs were clearly upregulated in dose-dependent manner. We therefore focused on these lncRNA, and investigated whether the cause of their upregulated expression levels in chemical stress conditions resulted from an increased transcription rate or a decreased decay rate. We determined the transcription rates and half-lives of LINC00152 and MALAT1 in the presence and absence of mercury II chloride and cycloheximide (Fig 3). Analyses of RNA transcription rates and half-lives were conducted by the EU pulse-labeling method [25]. EU is efficiently incorporated into the nascent RNAs in living cells. EU-labeled RNAs are separated from total RNAs by biotinylation of EU in a copper-catalyzed cycloaddition reaction, followed by purification on streptavidin magnetic beads. To assess the transcription rates, EU and chemicals were added to the culture medium and incubated for 2 h, and the amounts of isolated EU-labeled RNA were measured by RT-qPCR. To assess the half-lives, EU was firstly added to the culture medium and incubated for 2 h, and total RNAs were isolated from cells at sequential time points after removal of surplus EU from the culture medium, simultaneously with adding chemicals. Then, the amounts of isolated EU-labeled RNA were measured by RT-qPCR. When cells were treated with 100 μM mercury II chloride, the transcription rate of LINC00152 did not change, but the *t*_1/2_ of LINC00152 increased from 2.1 h to >6 h (Fig 3A). Similarly, when cells were treated with 100 μM cycloheximide, the transcription rate of MALAT1 did not change, but the *t*_1/2_ of MALAT1 increased from 1.8 h to >6 h (Fig 3B). These data indicate that the expression levels of LINC00152 and MALAT1 were elevated owing to reduced decay rates, rather than increased transcription rates, in response to chemicals.

### Expression of lncRNA-adjacent genes

**Fig 4:**
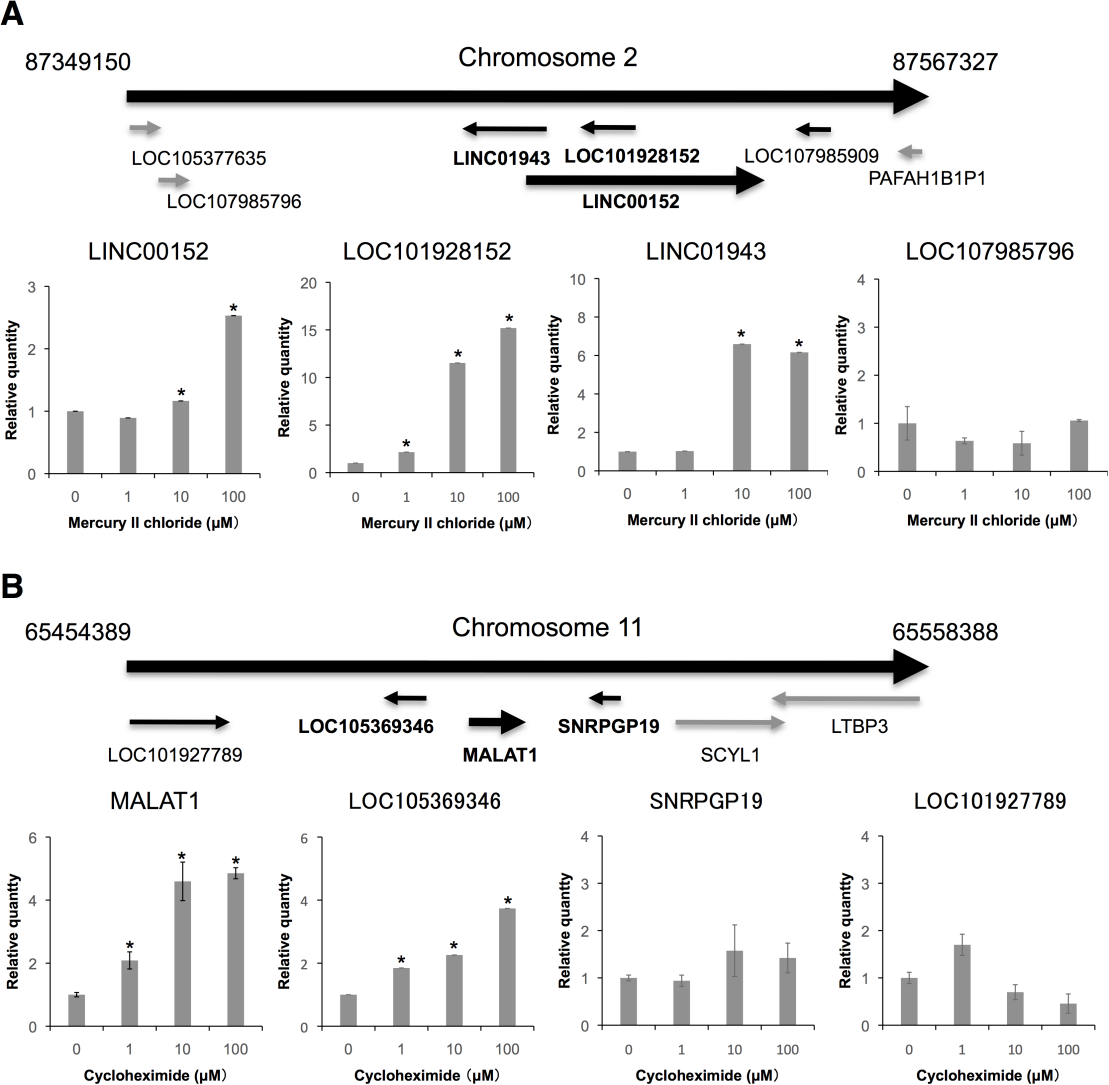
Alterations in LINC00152- and MALAT1-neighboring genes. Expression levels of LINC00152 (A) and MALAT1 (B) were determined by RT-qPCR. GAPDH, ACTB, HPRT1, and PGK1 were used for normalization. Values represent mean ± SD obtained from three independent experiments (*P < 0.05, Student’s t test).

Rapid induction of immediate-early genes in response to stimulation is known to be accompanied by co-upregulation of their neighboring genes [30]. We therefore analyzed the expression levels of lncRNA-adjacent genes in chemical-treated NSCs. When the cells were treated with 1, 10, or 100 μM mercury II chloride, LINC00152 and its very neighboring gene (LOC101928152) was simultaneously upregulated in a dose-dependent manner (Fig 4A). LINC00152 and LOC101928152 are overlapping; therefore, we designed the qPCR primers for LINC00152 and LOC101928152 with intron spanning. Long intergenic non-protein coding RNA 1943 (LINC01943) was also upregulated. In contrast, the expression levels of the other neighboring genes (LOC107985796 and LOC107985909) were not altered (Fig 4A) (LOC107985909 was not detected by RT-qPCR). When cells were treated with 1, 10, or 100 μM cycloheximide, MALAT1 and one of its very neighboring genes (LOC105369346) were upregulated in a dose-dependent manner; however, the expression levels of the other neighboring gene [LOC101927789, small nuclear ribonucleoprotein polypeptide G pseudogene 19 (SNRPGP19), SCY1 like pseudokinase 1 (SCYL1)] were not altered (Fig 4B and S1 Fig). These data suggest that LINC00152 and MALAT1, and their very neighboring genes, responded to chemical stresses.

### Regulation of neighboring genes by overexpression of lncRNAs

**Fig 5:**
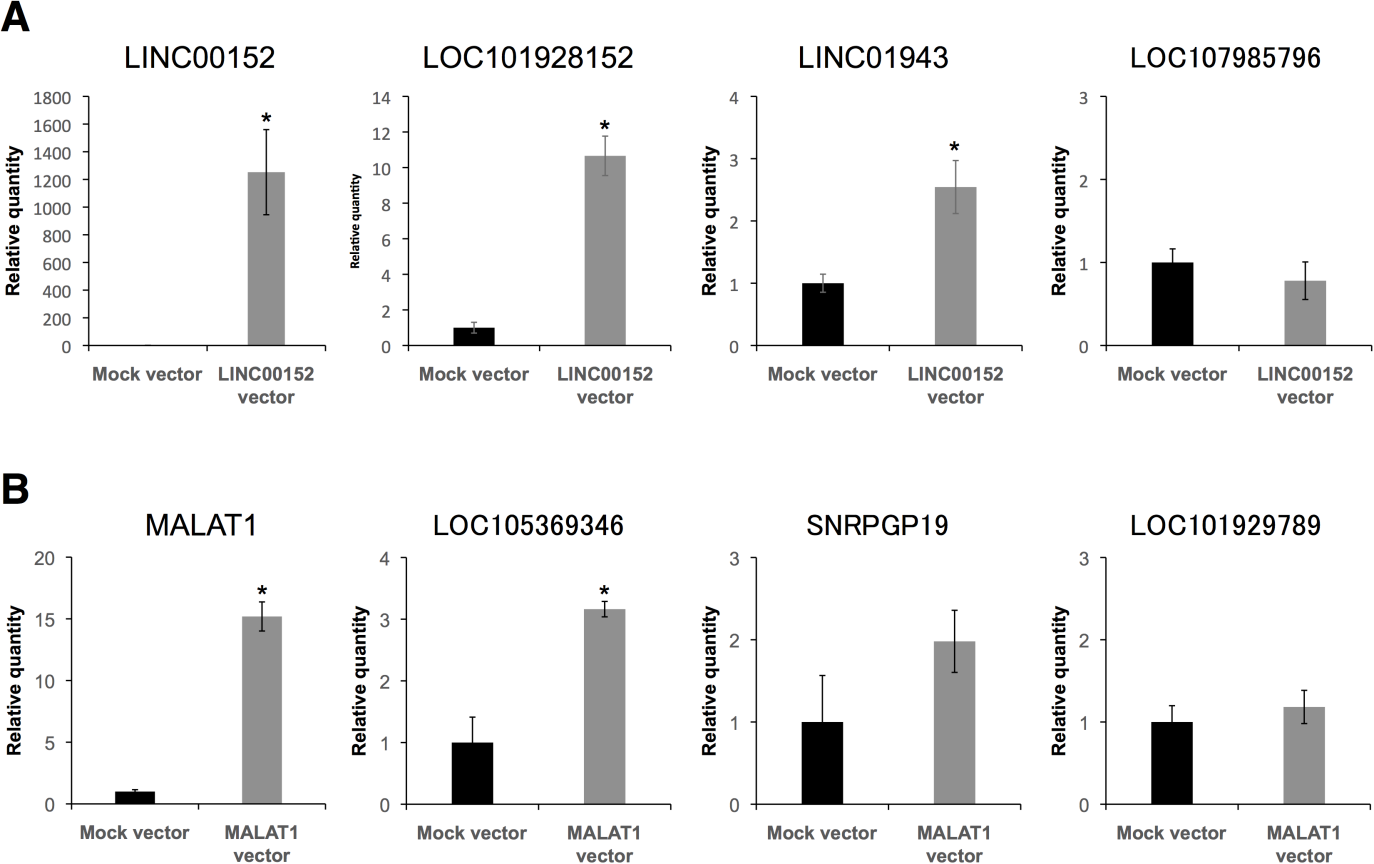
Overexpression of LINC00152 and MALAT1 increased expression levels of their neighboring genes. NSCs were treated with plasmid vectors as indicated. Expression levels of the indicated RNAs in control cells with mock vector (black bar) and in cells over-expressing (A) LINC00152 and (B) MALAT1 (gray bar) were determined by RT-qPCR. GAPDH, ACTB, HPRT1, and PGK1 were used for normalization. Values represent mean ± SD obtained from three independent experiments (*P < 0.05, Student’s t test).

To examine the extent to which LINC00152 and MALAT1 regulate their neighboring genes, we overexpressed LINC00152 and MALAT1 in NSCs (Fig 5). LOC101928152 and LINC01943 were upregulated by LINC00152 overexpression (Fig 5A). In contrast, LOC107985796 and LOC107985909 were not upregulated. Moreover, LOC105369346 was upregulated by MALAT1 overexpression, but as expected, SNRPGP19 and LOC101929789 were not upregulated (Fig 5B). These results are consistent with the findings described in the above section. This indicates that the respective neighboring genes can be regulated by LINC00152 or MALAT1 overexpression without chemical stress.

## Discussion

In this study, we identified four chemical-stress-responsive SLiTs (TUG1, GAS5, FAM222-AS1, and SNHG15) in NSCs. These four SLiTs were sensitive to chemical stresses and highly responsive to chemical stimulation. Traditional mRNA biomarkers of stress also responded to chemical stresses; however, few of the mRNAs exhibited high expression levels, compared with the expression levels of the SLiTs. In HeLa-Tet-off cells, MALAT1 is a middle-lived non-coding transcript (*t*_1/2_ = ~7.6 h) [8], but our results show that the half-life of MALAT1 was 1.8 h in NSCs. This finding indicates MALAT1 can be classified as a SLiT in NSCs. We also found that LINC00152 and MALAT1 expression levels were elevated in response to chemical stresses not because of an increase in transcription rate, but because of a reduction in decay rate. The fold changes of LINC00152 upon mercury II chloride treatment was ~10 in Fig 2B but only 3 in Fig 4A. The fold changes of MALAT1 upon cycloheximde was over 8 in Fig 2C but less than 6 in Fig 4B. These differences result from run-to-run variation. These results indicate that RNA degradation pathways are interrupted. In this paper, the RNA degradation pathways of LINC00152 and MALAT1 were not elucidated, but previous papers reported that several RNA degradation pathways such as nonsense-mediated decay (NMD) [31], no-go-decay [32], nuclear exosome targeting (NEXT) [33], and poly(A) tail exosome targeting (PAXT) [34]. We consider that the revealing of RNA degradation pathways of LINC00152 and MALAT1 is questions of further research. We also found that levels of LINC00152 and MALAT1 and of transcripts of their neighboring genes fluctuated in response to chemical stresses. Specifically, LINC00152 and MALAT1 modulated the expression levels of their neighboring genes.

LINC00152 has been shown to act as an oncogene, based on the finding that LINC00152 knockdown inhibits cell proliferation and colony formation, promotes cell cycle arrest at G1 phase, triggers late apoptosis, reduces epithelial to mesenchymal transition, and suppresses cell migration and invasion [26]. LINC00152 knockdown also suppresses cell proliferation and tumor growth, and LINC00152 has been shown to directly bind with epidermal growth factor receptor (EGFR), thereby activating phosphoinositide 3-kinase (PI3K)/serine/threonine kinase (AKT) signaling [27]. LINC00152 is also involved in hepatocellular carcinoma oncogenesis via activation of the mechanistic target of rapamycin (mTOR) signaling pathway [28].

MALAT1 was originally identified as a transcript showing significant expression in individuals exhibiting high risk for metastasis of non-small cell lung tumors [25], and subsequently showed broad expression in normal human and mouse tissues and was found to be overexpressed in many human carcinomas, including those of the breast, pancreas, lung, colon, prostate, and liver [16,25,29]. In mice, among a small number of Malat1 neighboring genes in *cis* with significant changes in expression levels and in adult Malat1 knockout mice [35]. Moreover, MALAT1 in human cells localizes to hundreds of genomic sites and interact with the Polycomb repressive complex (PRC1) subunit CBX4 and helps to modulate growth-control gene localization in the nucleus [36]. These previous reports provide supporting our evidence that MALAT1 regulates cis-gene regions. Considering our results and these previous findings together, we propose a hypothesis whereby upregulation of LINC00152 and MALAT1 by chemical stresses, as observed in the present study, induced the transformation of cells described previously.

The functions of the SLiTs upregulated by chemical stresses in this study (HCG18, LOC550112, GABPB1-AS1, LINC01184, LINC00472_v2, FAM222-AS1, LINC0541471_v1, LINC0541471_v2, and SNHG15) remain unknown. However, we have demonstrated that SLiTs have potential as surrogate indicators of general or specific cell stress in NSCs. Furthermore, we have found that LINC00152 and MALAT1 might modulate the expressions of their neighboring genes in response to chemical stresses. We believe that this study will help to bridge the knowledge gap between digital genomic information and cellular function.

## Supporting information

S1 Fig Alterations in MALAT1-neighboring gene. Expression levels of SCYL1 were determined by RT-qPCR. GAPDH, ACTB, HPRT1, and PGK1 were used for normalization. Values represent mean ± SD obtained from three independent experiments.
(PDF)

S1 Table mRNA biomarker list. (PDF)

S2 Table Abbreviated terms in this study. (PDF)

S3 Table Primer pairs for RT-qPCR. (PDF)

S4 Table Alterations in mRNA and lncRNA expression levels in NSCs in response to hydrogen peroxide. (PDF)

S5 Table Alterations in mRNA and lncRNA expression levels in NSCs in response to mercury II chloride. (PDF)

S6 Table Alterations in mRNA and lncRNA expression levels in NSCs in response to cycloheximide. (PDF)

S7 Table Alterations in mRNA and lncRNA expression levels in NSCs in response to zinc chloride. (PDF)
