# Supplementary material for: Short-lived long non-coding RNAs as surrogate indicators for chemical exposure and LINC00152 and MALAT1 modulate their neighboring genes
Source: PLoS One. 2017 Jul 18;12(7):e0181628. doi: 10.1371/journal.pone.0181628 (PMC5515456; doi:10.1371/journal.pone.0181628)
Supplement: S1 Fig — Expression levels of SCYL1 were determined by RT-qPCR. GAPDH, ACTB, HPRT1, and PGK1 were used for normalization. Values represent mean ± SD obtained from three independent experiments. (PDF) [file pone.0181628.s001.pdf]

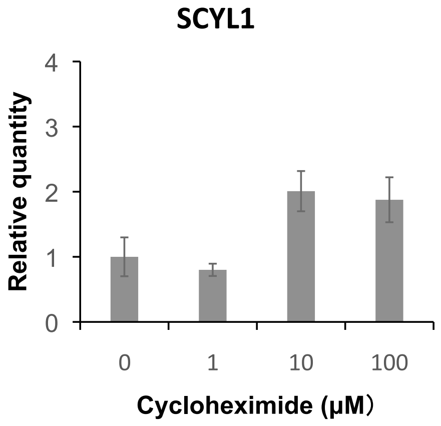

S1 Fig. Alterations in MALAT1-neighboring gene. Expression levels of SCYL1 were determined by RT-qPCR. GAPDH, ACTB, HPRT1, and PGK1 were used for normalization. Values represent mean ± SD obtained from three independent experiments.
