# Supplementary material for: Short-lived long non-coding RNAs as surrogate indicators for chemical exposure and LINC00152 and MALAT1 modulate their neighboring genes
Source: PLoS One. 2017 Jul 18;12(7):e0181628. doi: 10.1371/journal.pone.0181628 (PMC5515456; doi:10.1371/journal.pone.0181628)
Supplement: S1 Table — (PDF) [file pone.0181628.s002.pdf]

S1 Table. mRNA biomarkers list

Gene	Function of Stress
SOX1	Differentiation marker
POU5F1	Differentiation marker
NFKB1	Oxidative stress
JUN	Oxidative stress
HIF1A	Oxidative stress
PPP1R15A	DNA damage
GADD45A	DNA damage
DDIT3	DNA damage
TP53	DNA damage
CDKN1A	DNA damage
TP53I3	DNA damage
HSPA4	Heat shock response
HSP90AA1	Heat shock response
HSF1	Heat shock response
ATF3	ER stress Response
ERO1A	ER stress Response
BBC3	ER stress Response
ARNT	Hypoxia Inducible Factor
MTF1	Heavy Metal Stress
