# Supplementary material for: Short-lived long non-coding RNAs as surrogate indicators for chemical exposure and LINC00152 and MALAT1 modulate their neighboring genes
Source: PLoS One. 2017 Jul 18;12(7):e0181628. doi: 10.1371/journal.pone.0181628 (PMC5515456; doi:10.1371/journal.pone.0181628)
Supplement: S2 Table — (PDF) [file pone.0181628.s003.pdf]

S2 Table. Abbreviated terms in this study

Gene name	Formal name
"mRNA"	
GAPDH	glyceraldehyde-3-phosphate dehydrogenase
ACTB	actin beta
HPRT1	hypoxanthine phosphoribosyltransferase 1
PGK1	phosphoglycerate kinase 1
SOX1	SRY-box 1
POU5F1	POU class 5 homeobox 1
NFKB1	nuclear factor kappa B subunit 1
JUN	Jun proto-oncogene, AP-1 transcription factor subunit
HIF1A	hypoxia inducible factor 1 alpha subunit
PPP1R15A	protein phosphatase 1 regulatory subunit 15A
GADD45A	growth arrest and DNA damage inducible alpha
DDIT3	DNA damage inducible transcript 3
TP53	tumor protein p53
CDKN1A	cyclin dependent kinase inhibitor 1A
TP53I3	tumor protein p53 inducible protein 3
HSPA4	heat shock protein family A (Hsp70) member 4
HSP90AA1	heat shock protein 90 alpha family class A member 1
HSF1	heat shock transcription factor 1
ATF3	activating transcription factor 3
ERO1A	endoplasmic reticulum oxidoreductase 1 alpha
BBC3	BCL2 binding component 3
ARNT	aryl hydrocarbon receptor nuclear translocator
MTF1	metal regulatory transcription factor 1
"ncRNA"	
CDKN2B-AS1	CDKN2B antisense RNA 1
HOTAIR	HOX transcript antisense RNA
TUG1	taurine up-regulated 1
GAS5	growth arrest specific 5
MIR22HG	MIR22 host gene
LINC-PINT	long intergenic non-protein coding RNA, p53 induced transcript
KMT2E-AS1	KMT2E antisense RNA 1
LINC00667	long intergenic non-protein coding RNA 667
HCG18	HLA complex group 18
LOC550112	LOC550112
LINC00662	long intergenic non-protein coding RNA 662
GABPB1-AS1	GABPB1 antisense RNA 1
LINC01184	long intergenic non-protein coding RNA 1184
TTN-AS1	TTN antisense RNA 1
LINC01137	long intergenic non-protein coding RNA 1137
LINC00473_v1	long intergenic non-protein coding RNA 473 variant 1
LINC00473_v2	long intergenic non-protein coding RNA 473 variant 2
FAM222A-AS1	FAM222A antisense RNA 152
LINC00152	long intergenic non-protein coding RNA 152
LINC0541471_v1	long intergenic non-protein coding RNA 54171 variant 1
LINC0541471_v2	long intergenic non-protein coding RNA 54171 variant 2
IDI2-AS1	IDI2 antisense RNA 1
SNHG15	small nucleolar RNA host gene 15
ZFP91-CNTF	ZFP91-CNTF readthrough
MALAT1	metastasis associated lung adenocarcinoma transcript 1
NEAT1_v1	nuclear paraspeckle assembly transcript 1 variant 1
NEAT1_v2	nuclear paraspeckle assembly transcript 1 variant 2
