# Supplementary material for: Short-lived long non-coding RNAs as surrogate indicators for chemical exposure and LINC00152 and MALAT1 modulate their neighboring genes
Source: PLoS One. 2017 Jul 18;12(7):e0181628. doi: 10.1371/journal.pone.0181628 (PMC5515456; doi:10.1371/journal.pone.0181628)
Supplement: S3 Table — (PDF) [file pone.0181628.s004.pdf]

S3 Table. Primer pairs for RT-qPCR

Name	Sence sequece (5'-3')	Antisense sequece (5'-3')
"mRNA"		
GAPDH	GCACCGTCAAGGCTGAGAAC	TGGTGAAGACGCCAGTGGA
ACTB	CCAACCGCGAGAAGATGA	CCAGAGGCGTACAGGGATAG
HPRT1	TGACCTTGATTTATTTTGCATACC	CGAGCAAGACGTTCAGTCCT
PGK1	CTGTGGCTTCTGGCATACCT	CGAGTGACAGCCTCAGCATA
SOX1	ACCAGGCCATGGATGAAG	CTTAATTGCTGGGGAATTGG
POU5F1	ATCAAGCAGCGACTATGCAC	CCAGAGGAAAGGACACTGGT
NFKB1	CTGGCAGCTCTTCTCAAAGC	TCCAGGTCATAGAGAGGCTCA
JUN	CCAAAGGATAGTGCGATGTTT	CTGTCCCTCTCCACTGCAAC
HIF1A	TTTTTCAAGCAGTAGGAATTGGA	GTGATGTAGTAGCTGCATGATCG
PPP1R15A	GCTTCTGGCAGACCGAAC	GTAGCCTGATGGGGTGCTT
GADD45A	TTTGCAATATGACTTTGGAGGA	CATCCCCCACCTTTCCAT
DDIT3	CAGAGCTGGAACCTGAGGAG	CCTGCCGTTTAAAATTTTTGG
TP53	CATGAGCGCTGCTCAGATAG	ACACGCAAATTTCCTTCCAC
CDKN1A	CTGGTACCCTCCTGGCTCTT	CCCAGTGCAGGTCAGAGG
TP53I3	AATGCTTTCACGGAGCAAATTC	TTCGGTCACTGGGTAGATTCT
HSPA4	CAGCAGACACCAGCAGAAAA	CCTTGGATCCAGCTTGAGAG
HSP90AA1	GGGCAACACCTCTACAAGGA	CTTGGGTCTGGGTTTCCTC
HSF1	CAAGCTGTGGACCCTCGT	TCGAACACGTGGAAGCTGT
ATF3	AAGGATTTTCAGCACCTTGC	GATGGCAGAAGCACTCACTTC
ERO1A	GGAGACAGCGGCACAGAG	CAATGGTTTCAACATCACAGGT
BBC3	GACCTCAACGCACAGTACGAG	AGGAGTCCCATGATGAGATTGT
ARNT	CTACCCGCTCAGGCTTTTC	CACCAAACTGGGAAGTACGAG
MTF1	TGAATTGCGAGAAAATTCCA	GCTGGTGAAATTGTGCTGAG
″ncRNA″		
CDKN2B-AS1	TTGTTAGAAACCAGGCTGCAC	TTCTCTCTTTCTGTGGTTTCTCAAT
HOTAIR	CAGTGGGGAACTCTGACTCG	GTGCCTGGTGCTCTCTTACC
TUG1	CCAGACCCTCAGTGCAAACT	AATCAGGAGGCACAGGACA
GAS5	CTTGCCTGGACCAGCTTAAT	CAAGCCGACTCTCCATACCT
MIR22HG	CGGACGCAGTGATTTGCT	GCTTTAGCTGGGTCAGGACA
LINC-PINT	GGGATAATTTGCCATCTGGA	CCGTTTCTTCCATTTTCCTCT
KMT2E-AS1	CAGCCAAAATCTGGCCTACT	GTCCAGCAATCATTTTCTCGT
LINC00667	AGTTTGCGCCTTTTGGTCT	GGCCATGTGCAAAGGATTT
HCG18	CTGCCTTCTAGGGGCTCACT	ACATGCTCCACCAACTTTCA
LOC550112	CTGAAATCAGAGCCTGCACA	TCAAGGACCTGGAAATGACC
LINC00662	GTTTGATTTCTCGCAGACCAG	GCGAGGTCTAACCCAGGTG
GABPB1-AS1	AGGGAAAGAAAATATGCCATTTCTA	ATCATTCCGCCGCTTTCT
LINC01184	GCAATTATCACGGGAAACCTAT	AAATCTTATCTGCTTCCCTATTTGTAA
TTN-AS1	TCCTTAGGCATCACCTAGCC	GATGGAGGAAGTAGAGTCATTGG
LINC01137	CACTCCCTAACCCGTGTCC	TCCATCAAAGCAGCCAGAC
LINC00473_v1	TATGCGCGTCAGCATACTTT	TGTCCTGTGCCTCCCTGT
LINC00473_v2	TATGCGCGTCAGCATACTTT	TCTCCCAAAGCACAACGAG
FAM222A-AS1	CAACATGGAAATGGAGACCA	CTTCCGGGATCCCAGTGT
LINC00152	CGTGCCTGTCTTCAGATCTTC	TCATCTCCCAGTTATTCAAGGAG
LINC0541471_v1	CAGATCTTCACAGCACAGTTCC	TGCTGATCCACTTTGCTTGT
LINC0541471_v2	CACCAGCCTCTCCCTGAA	TTCGATCAAGTGTGTCATAGAGC
IDI2-AS1	GTGTTAAACAAGACAACGCTGAA	AAGAGCGCTGGAAAAACCTT
SNHG15	GCAACTCCTTTGCAAGATGC	CTCAAGGAGGGACCTCAGC
ZFP91-CNTF	TTGTTCATACTTGGCGGTGA	GGCGGGCCTAATCATTTT
MALAT1	GCTGTGGAGTTCTTAAATATCAACC	TTCTCAATCCTGAAATCCCCTA
NEAT1_v1	TTGTTCCAGAGCCCATGAT	TGAAAACCTTTACCCCAGGA
NEAT1_v2	GATCTTTTCCACCCCAAGAGTACATAA	CTCACACAAACACAGATTCCACAAC
