# Supplementary material for: Short-lived long non-coding RNAs as surrogate indicators for chemical exposure and LINC00152 and MALAT1 modulate their neighboring genes
Source: PLoS One. 2017 Jul 18;12(7):e0181628. doi: 10.1371/journal.pone.0181628 (PMC5515456; doi:10.1371/journal.pone.0181628)
Supplement: S4 Table — (PDF) [file pone.0181628.s005.pdf]

S4 Table. Alterations in mRNA and lncRNA expression levels in NSCs in response to hydrogen peroxide

Gene	Mean (Exposure/Control)	SD	*P*-value
SOX1	0.0129	0.0001	< 0.05
POU5F1	0.0028	0.0001	< 0.05
NFKB1	0.8643	0.0250	< 0.05
JUN	0.2030	0.3369	< 0.05
HIF1A	3.8692	0.0096	< 0.05
PPP1R15A	0.0412	0.0864	< 0.05
GADD45A	0.8076	0.0770	< 0.05
DDIT3	0.6807	0.0785	< 0.05
TP53	6.8266	0.4710	< 0.05
CDKN1A	1.9200	0.4209	< 0.05
TP53I3	1.0734	0.0018	< 0.05
HSPA4	3.4337	0.0035	< 0.05
HSP90AA1	0.0185	0.0035	< 0.05
HSF1	0.2728	0.0118	< 0.05
ATF3	0.0124	0.0023	< 0.05
ERO1A	3.9314	0.1697	< 0.05
BBC3	0.4536	0.1198	< 0.05
ARNT	8.5513	0.0610	< 0.05
MTF1	1.8965	0.0115	< 0.05
CDKN2B-AS1	0.0011	0.0002	< 0.05
HOTAIR	0.0007	0.0003	< 0.05
TUG1	8.4886	0.2396	< 0.05
GAS5	7.5617	0.0595	< 0.05
MIR22HG	0.0013	0.0015	< 0.05
LINC-PINT	0.0025	0.0006	< 0.05
KMT2E-AS1	0.2721	0.0071	< 0.05
LINC00667	0.9344	0.0174	< 0.05
HCG18	7.1266	0.3656	< 0.05
LOC550112	4.4617	0.2805	< 0.05
LINC00662	1.1328	0.2117	0.39
GABPB1-AS1	6.3896	0.0709	< 0.05
LINC01184	7.7228	0.2802	< 0.05
TTN-AS1	2.5195	0.1003	< 0.05
LINC01137	0.0595	0.0093	0.45
LINC00473_v1	0.0340	0.0223	< 0.05
LINC00473_v2	0.5135	0.4692	< 0.05
FAM222A-AS1	7.1735	0.4098	< 0.05
LINC00152	0.6233	0.0465	< 0.05
LINC0541471_v1	1.1835	0.0826	< 0.05
LINC0541471_v2	0.4487	0.4151	< 0.05
IDI2-AS1	0.0222	0.0062	< 0.05
SNHG15	8.8213	0.1424	< 0.05
ZFP91-CNTF	0.0032	0.0001	< 0.05
MALAT1	1.9982	0.3817	< 0.05
NEAT1_v1	0.0000	0.0000	0.26
NEAT1_v2	0.0264	0.0009	< 0.05
