# Supplementary material for: Short-lived long non-coding RNAs as surrogate indicators for chemical exposure and LINC00152 and MALAT1 modulate their neighboring genes
Source: PLoS One. 2017 Jul 18;12(7):e0181628. doi: 10.1371/journal.pone.0181628 (PMC5515456; doi:10.1371/journal.pone.0181628)
Supplement: S5 Table — (PDF) [file pone.0181628.s006.pdf]

S5 Table. Alterations in mRNA and lncRNA expression levels in NSCs in response to mercury II chloride

Gene	Mean (Exposure/Control)	SD	*P*-value
SOX1	0.0068	0.0003	< 0.05
POU5F1	0.0139	0.0005	< 0.05
NFKB1	0.3838	0.0164	< 0.05
JUN	8.7642	1.0152	< 0.05
HIF1A	2.1473	0.1837	< 0.05
PPP1R15A	1.8925	0.0677	< 0.05
GADD45A	3.7185	0.2204	< 0.05
DDIT3	3.0090	0.1759	< 0.05
TP53	2.2255	0.1802	< 0.05
CDKN1A	11.1951	0.8612	< 0.05
TP53I3	0.2868	0.0057	< 0.05
HSPA4	5.9282	0.0012	< 0.05
HSP90AA1	0.0235	0.0124	0.11
HSF1	0.2694	0.0082	< 0.05
ATF3	2.6108	0.1549	< 0.05
ERO1A	0.9295	0.0596	< 0.05
BBC3	0.8568	0.0801	< 0.05
ARNT	3.4225	0.1254	< 0.05
MTF1	4.4830	0.1894	< 0.05
CDKN2B-AS1	0.0000	0.0000	< 0.05
HOTAIR	0.0117	0.0016	< 0.05
TUG1	6.9834	0.3605	< 0.05
GAS5	4.9620	0.2170	< 0.05
MIR22HG	0.0851	0.0055	< 0.05
LINC-PINT	0.0291	0.0037	< 0.05
KMT2E-AS1	0.2736	0.0159	< 0.05
LINC00667	0.6613	0.0313	< 0.05
HCG18	2.2017	0.1593	< 0.05
LOC550112	2.3558	0.1928	< 0.05
LINC00662	0.6256	0.3618	0.32
GABPB1-AS1	5.4931	0.2504	< 0.05
LINC01184	2.3806	0.2292	< 0.05
TTN-AS1	2.5263	0.1473	< 0.05
LINC01137	0.2907	0.0168	< 0.05
LINC00473_v1	0.0383	0.0125	< 0.05
LINC00473_v2	2.5873	0.1301	< 0.05
FAM222A-AS1	26.4457	1.7687	< 0.05
LINC00152	6.3849	0.4670	< 0.05
LINC0541471_v1	17.9862	0.9079	< 0.05
LINC0541471_v2	4.1788	0.2858	< 0.05
IDI2-AS1	0.1413	0.0055	< 0.05
SNHG15	4.7449	0.2041	< 0.05
ZFP91-CNTF	0.0022	0.0001	0.34
MALAT1	1.9152	0.1362	< 0.05
NEAT1_v1	0.0001	0.0000	< 0.05
NEAT1_v2	0.3980	0.0259	< 0.05
