# Supplementary material for: Short-lived long non-coding RNAs as surrogate indicators for chemical exposure and LINC00152 and MALAT1 modulate their neighboring genes
Source: PLoS One. 2017 Jul 18;12(7):e0181628. doi: 10.1371/journal.pone.0181628 (PMC5515456; doi:10.1371/journal.pone.0181628)
Supplement: S6 Table — (PDF) [file pone.0181628.s007.pdf]

S6 Table. Alterations in mRNA and lncRNA expression levels in NSCs in response to cycloheximide

Gene	Mean (Exposure/Control)	SD	*P*-value
SOX1	0.0015	0.0001	＜ 0.05
POU5F1	0.0026	0.0001	＜ 0.05
NFKB1	1.1205	0.0250	＜ 0.05
JUN	4.0273	0.3369	＜ 0.05
HIF1A	0.0991	0.0096	＜ 0.05
PPP1R15A	1.0305	0.0864	＜ 0.05
GADD45A	2.8221	0.0770	＜ 0.05
DDIT3	0.9482	0.0785	＜ 0.05
TP53	6.6769	0.4710	＜ 0.05
CDKN1A	4.8258	0.4209	＜ 0.05
TP53I3	0.3557	0.1795	0.307
HSPA4	5.9737	0.0035	＜ 0.05
HSP90AA1	0.0507	0.0035	＜ 0.05
HSF1	0.2690	0.0118	＜ 0.05
ATF3	0.0138	0.0023	＜ 0.05
ERO1A	1.6867	0.1697	＜ 0.05
BBC3	1.9203	0.1198	＜ 0.05
ARNT	2.3097	0.0610	＜ 0.05
MTF1	0.9669	0.0115	＜ 0.05
CDKN2B-AS1	0.0020	0.0002	＜ 0.05
HOTAIR	0.0023	0.0003	＜ 0.05
TUG1	6.5154	0.2396	＜ 0.05
GAS5	2.7227	0.0595	＜ 0.05
MIR22HG	0.0294	0.0015	＜ 0.05
LINC-PINT	0.0101	0.0006	＜ 0.05
KMT2E-AS1	0.0945	0.0071	0.065
LINC00667	0.7910	0.0174	＜ 0.05
HCG18	4.6366	0.3656	＜ 0.05
LOC550112	4.4596	0.2805	＜ 0.05
LINC00662	0.3323	0.2117	0.394
GABPB1-AS1	1.7261	0.0709	＜ 0.05
LINC01184	3.4119	0.2802	＜ 0.05
TTN-AS1	1.3750	0.1003	0.069
LINC01137	0.0203	0.0093	0.051
LINC00473_v1	0.2957	0.0223	＜ 0.05
LINC00473_v2	4.3271	0.4692	＜ 0.05
FAM222A-AS1	6.4233	0.4098	＜ 0.05
LINC00152	0.5355	0.0465	＜ 0.05
LINC0541471_v1	6.3338	0.0826	＜ 0.05
LINC0541471_v2	5.4838	0.4151	＜ 0.05
IDI2-AS1	0.1117	0.0062	＜ 0.05
SNHG15	6.0080	0.1424	＜ 0.05
ZFP91-CNTF	0.0023	0.0001	＜ 0.05
MALAT1	5.9783	0.3817	＜ 0.05
NEAT1_v1	0.0000	0.0000	＜ 0.05
NEAT1_v2	0.0338	0.0009	＜ 0.05
