# Supplementary material for: Short-lived long non-coding RNAs as surrogate indicators for chemical exposure and LINC00152 and MALAT1 modulate their neighboring genes
Source: PLoS One. 2017 Jul 18;12(7):e0181628. doi: 10.1371/journal.pone.0181628 (PMC5515456; doi:10.1371/journal.pone.0181628)
Supplement: S7 Table — (PDF) [file pone.0181628.s008.pdf]

S7 Table. Alterations in mRNA and lncRNA expression levels in NSCs in response to zinc chloride

Gene	Mean (Exposure/Control)	SD	*P*-value
SOX1	0.0034	0.0003	< 0.05
POU5F1	0.0009	0.0001	< 0.05
NFKB1	0.1641	0.0043	< 0.05
JUN	3.6025	0.5233	< 0.05
HIF1A	1.0854	0.1179	< 0.05
PPP1R15A	0.0119	0.0016	< 0.05
GADD45A	0.1399	0.0079	< 0.05
DDIT3	0.4891	0.0822	0.089
TP53	1.5572	0.1543	< 0.05
CDKN1A	0.8442	0.1194	< 0.05
TP53I3	0.1947	0.0090	< 0.05
HSPA4	0.9488	0.0006	< 0.05
HSP90AA1	0.0052	0.0006	< 0.05
HSF1	0.0750	0.0099	< 0.05
ATF3	0.0031	0.0009	< 0.05
ERO1A	1.0519	0.1997	0.304
BBC3	0.1140	0.0264	< 0.05
ARNT	2.1091	0.1882	< 0.05
MTF1	0.3867	0.0257	< 0.05
CDKN2B-AS1	0.0002	0.0001	< 0.05
HOTAIR	0.0002	0.0001	< 0.05
TUG1	2.3703	0.2366	< 0.05
GAS5	2.4500	0.1477	< 0.05
MIR22HG	0.0003	0.0000	< 0.05
LINC-PINT	0.0011	0.0002	0.208
KMT2E-AS1	0.0593	0.0065	0.23
LINC00667	0.2465	0.0235	< 0.05
HCG18	2.6216	0.3966	< 0.05
LOC550112	1.3980	0.1575	< 0.05
LINC00662	0.3308	0.0333	< 0.05
GABPB1-AS1	1.3761	0.1117	0.184
LINC01184	2.4922	0.3603	< 0.05
TTN-AS1	0.7577	0.0991	0.096
LINC01137	0.0102	0.0019	0.056
LINC00473_v1	0.0078	0.0042	< 0.05
LINC00473_v2	0.3215	0.0831	< 0.05
FAM222A-AS1	1.5095	0.2275	< 0.05
LINC00152	0.2077	0.0564	< 0.05
LINC0541471_v1	0.1779	0.0069	< 0.05
LINC0541471_v2	0.1057	0.0196	< 0.05
IDI2-AS1	0.0073	0.0008	< 0.05
SNHG15	2.9869	0.4029	< 0.05
ZFP91-CNTF	0.0006	0.0000	< 0.05
MALAT1	0.9143	0.1613	< 0.05
NEAT1_v1	0.0000	0.0000	< 0.05
NEAT1_v2	0.0060	0.0006	< 0.05
